# Generation Scotland - Using Electronic Health Records for Research.

**DOI:** 10.23889/ijpds.v7i3.2041

**Published:** 2022-08-25

**Authors:** Archie Campbell, Robin Flaig, David Porteous, Cathie Sudlow

**Affiliations:** 1 University of Edinburgh

## Objectives

Generation Scotland (GS) is a family-based genetic epidemiology study of around 24,000 volunteers from 7000 families recruited across Scotland between 2006 and 2011 with follow-up through record linkage and re-contact. Broad consent was obtained for linkage to medical records for 98% of the cohort. Recruitment has recommenced in 2022.

## Approach

TParticipants completed a demographic, health and lifestyle questionnaire, provided biological samples, and underwent detailed clinical assessment. The samples, phenotype and genotype data form a resource with broad consent for health-related research of current and projected public health importance.

This has become a longitudinal dataset by linkage to routine NHS hospital, maternity, lab test, prescriptions, dentistry, mortality, cancer screening, GP data records, Covid-19 testing and vaccinations. Genome-wide association studies (GWAS) have been done on quantitative traits and biomarkers, with DNA methylation data and proteomics available for most of the cohort. Our “CovidLife” surveys collected data on the effects of the pandemic.

## Results

Researchers can use the linked datasets to ascertain prevalent and incident disease cases and controls to test a wide range of research hypotheses. They can also recruit participants to new studies, including recall by genotype, utilising the NHS Scotland Community Health Index (CHI) Register for current contact details. We have established and validated electronic health record linkage, overcoming technical and governance issues in the process. Using consented data avoids some limitations of safe havens for analysis.

GS is a contributor to major international consortia, with collaborators from many institutions worldwide, both academic and commercial. New recruits are now asked to give consent to linkage to other administrative data, and reuse of samples from routine NHS tests for medical research.

## Conclusion

GS is now extending linkages to include radiology and health-relevant administrative data (e.g. education); and reopening recruitment to double the cohort size, including teenagers age 12+, collecting new data online and using remote sample collection.

GS resources are available to academic and commercial researchers through a managed access process (www.generationscotland.org).

